# Association between serum homocysteine levels and advanced hepatic fibrosis in alcohol-related liver disease: A cross-sectional study of NHANES

**DOI:** 10.1097/MD.0000000000043395

**Published:** 2025-07-25

**Authors:** Cheng Ma, Xiaoqian Zhang, Wenxin Zhang, Jianzhou Duan, Hui Yang

**Affiliations:** a Department of Infectious Diseases, Heping Hospital Affiliated to Changzhi Medical College, Changzhi, Shanxi Province, China; b First Clinical Medical College, Shanxi Medical University, Taiyuan, Shanxi Province, China; c Department of Infectious Diseases, The First Hospital of Shanxi Medical University, Taiyuan, Shanxi Province, China; d Department of Infectious Diseases, Xi’an Eighth Hospital, Xi’an, Shaanxi Province, China.

**Keywords:** advanced hepatic fibrosis, alcohol-related liver disease, cross-sectional study, homocysteine, NHANES

## Abstract

Homocysteine (Hcy) can induce liver cell damage, but its relationship with alcohol-related liver disease (ALD) has rarely been reported. This study aimed to investigate the association between serum Hcy levels and advanced hepatic fibrosis in patients with ALD. We included 10,033 participants from the 1999 to 2006 National Health and Nutrition Examination Survey. Four hundred ninety six individuals with excessive alcohol consumption, elevated liver enzymes, and no other chronic liver disease were identified as ALD. Fibrosis-4 index, aspartate aminotransferase to platelet ratio index, and Frons index were used as noninvasive indicators for assessing the extent of liver fibrosis. Weighted multivariate logistic regression was used to analyze the correlation between serum Hcy levels and advanced hepatic fibrosis in ALD participants. Compared to non-alcoholic liver disease, ALD participants had higher serum Hcy levels (*P* < .001). In weighted multivariable-adjusted logistic regression models, we observed a positive correlation between serum Hcy levels and the risk of advanced hepatic fibrosis in ALD (odds ratio [OR] = 1.07, 95% confidence interval [CI], 1.01–1.12, *P* < .05), and the highest tertile of Hcy was significantly associated with an increased risk of advanced hepatic fibrosis (OR = 3.36, 95% CI, 1.34–8.43, *P* < .05). In subgroup analyses stratified by gender, physical activity, and body mass index, this association remained significant in men (OR = 1.07, 95% CI, 1.01–1.13, *P* = .026), vigorously physically active (OR = 1.46, 95% CI, 1.06–2.01, *P* = .027), and obese participants (OR = 1.36, 95% CI, 1.10–1.67, *P* = .008). In ALD participants, the area under the working characteristic curve of Hcy for advanced hepatic fibrosis was 0.686 (95% CI, 0.639–0.733). Serum Hcy levels were independently associated with an increased risk of advanced hepatic fibrosis in ALD, especially among men, vigorously physically active, and obese populations. This study supports the predictive value of Hcy for advanced hepatic fibrosis and suggests that Hcy may become a therapeutic entry point for ALD.

## 1. Introduction

Alcohol-related liver disease (ALD) is a disease of the liver caused by long-term heavy alcohol consumption, with a spectrum of diseases ranging from simple steatosis to more severe liver injury, including alcoholic hepatitis, fibrosis, cirrhosis, and superimposed hepatocellular carcinoma.^[1]^ In Western and industrialized countries, ALD has replaced viral hepatitis as the main cause of advanced hepatic fibrosis and cirrhosis,^[2,3]^ and due to the increase in alcohol abuse in recent years, some developing countries and regions are gradually experiencing the etiologic shift from infectious to alcoholic liver disease.^[4,5]^ Hepatic fibrosis is a dynamic pathological process that is characterized by oxidative stress and inflammatory response inducing activation of hepatic stellate cells (HSCs) and leading to the net accumulation of extracellular matrix in the progression of chronic liver disease.^[6,7]^ Hepatic fibrosis is a necessary pathway for developing early liver disease into cirrhosis. Recent epidemiological research studies have shown that alcoholic cirrhosis patients have a higher frequency of readmission and a higher need for liver transplantation than other types of cirrhosis, and their medical expenses have almost doubled.^[8,9]^ The national disease burden is growing. Therefore, developing sensitive predictive biomarkers for identifying advanced hepatic fibrosis in ALD patients is particularly important.

Homocysteine (Hcy) is an intermediate product in methionine metabolism and a nonprotein amino acid in the body. Elevated serum levels of Hcy have been shown to be closely related to the occurrence of a variety of diseases including cardiovascular disease, diabetes, kidney diseases, bone tissue damage, neurodegenerative disorders, and neural tube defects,^[10,11]^ Hcy has become a widely used biochemical indicator in clinical practice. The liver is the primary site of methionine metabolism and transsulfuration pathways, and hepatic parenchymal cell injury due to various etiologies will cause impaired Hcy metabolism.^[12]^ However, there is still limited research on the correlation between Hcy levels and liver cell injury. Existing studies remain insufficient evidence on the independent role of Hcy in ALD disease progression. Currently, the fibrosis-4 index (FIB-4), aspartate aminotransferase to platelet ratio index (APRI), and Frons index have been considered as noninvasive tools in clinical practice to assess the extent of fibrosis in chronic liver disease.^[13,14]^ Our study aimed to reveal the association between serum Hcy and advanced hepatic fibrosis in ALD patients, which provides a basis for subsequent studies.

## 2. Materials and methods

### 2.1. Study population and design

The National Health and Nutrition Examination Survey (NHANES) aims to evaluate the health and nutritional status of adults and children, this survey is an important way to understand and improve the health of the United States. Since 1999, the survey has adopted a complex, cross-sectional, multistage, sustained, and large base sampling design. The NHANES sample represents the entire U.S. population. NHANES combines data from interviews and physical examinations, including basic population information, dietary data, laboratory/imaging tests, and questionnaire interviews. NHANES survey data are available on the internet (http://www.cdc.gov/nchs/nhanes.htm). The National Center for Health Statistics is responsible for protecting the confidentiality of all respondents. NHANES survey protocol was approved by the NCHS Ethics Review Board and documented consent was obtained from participants.

**Figure 1.**
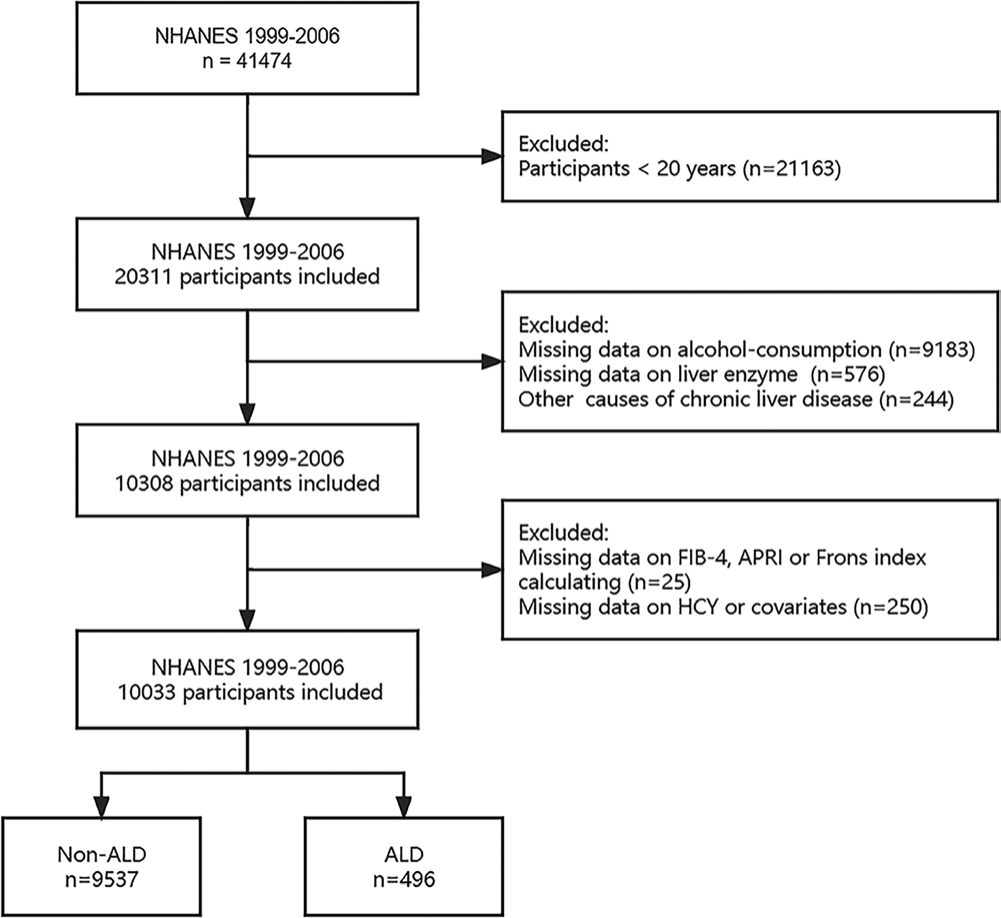
The flowchart of this study. ALD = alcohol-related liver disease, Hcy = homocysteine, NHANES = National Health and Nutrition Examination Survey, Non-ALD = non-alcoholic liver disease.

We used the 1999 to 2006 NHANES dataset and screened 20,311 participants aged ≥20 years. We conducted demographic data and health-related information interviews with them, and blood specimens were collected at the mobile examination centers for standard biochemistry profiles, glycohemoglobin, blood cholesterol, and other tests. In this study, we excluded participants with incomplete data on alcohol consumption (n = 9183) and missing liver enzyme data (n = 576), as well as other causes of chronic liver diseases, including hepatitis C and hepatitis B infections (n = 244). We also excluded the participants whose liver fibrosis indicators FIB-4, APRI, and Frons index calculating were missing (n = 25), and missing serum Hcy data and key covariates data were further excluded (n = 250), including age, gender, race, education level, physical activity, body mass index (BMI), smoking status, alcohol consumption, history of diabetes, serum folate, vitamin B_12_, total cholesterol, and high-density lipoprotein levels. Finally, 10,033 participants were included in this study for analysis (Fig. 1).

### 2.2. Study definitions

#### 2.2.1. Alcohol-related liver disease (ALD)

All participants conducted a questionnaire survey on alcohol consumption and were asked: How often did you drink alcohol in the past 12 months? How many drinks did you have on average per day during your drinking days? Here, a drink means a 12-ounce beer, a 4 or 5-ounce glass of wine, or 1 or 1.5 ounces of liquor. Based on the self-reports of participants, we estimated the daily alcohol consumption: the average amount of drinks on drinking days × the number of drinking days in the past 12 mo/365. According to the definition of drinking levels by the National Institute on Alcohol Abuse and Alcoholism, excessive drinking is defined as an alcohol intake of 2 drinks or more per day for men and 1 drink or more per day for women. ALD was assumed to be participants who have excessive drinking and elevated liver enzyme levels (alanine aminotransferase > 30 U/L, aspartate aminotransferase > 33 U/L, alkaline phosphatase > 113 U/L, gamma-glutamyl transaminase > 36 U/L, total bilirubin > 1.3 mg/dL), and in the absence of hepatitis B (hepatitis B surface antigen positive) and hepatitis C infections (hepatitis C antibody or HCV RNA positive). The remaining participants were defined as non-alcoholic liver disease (non-ALD).

#### 2.2.2. Advanced hepatic fibrosis

We used FIB-4, APRI, and Frons index to evaluate advanced hepatic fibrosis. Intermediate-high risk of advanced hepatic fibrosis was defined as FIB-4 > 1.3, APRI > 1, and Frons index > 4.2, and used as thresholds for dichotomizing the degree of hepatic fibrosis in regression models.^[14,15]^ The calculation formulas were as follows:


$$\begin{array}{l} \;\;\;FIB\text{-}4=\frac{age\;\;\;\left(years\right)\;\times \;AST\;\left(U/L\right)}{PLT\;\left(1000\;cells/\;\mu \;L\right)\;\times \;\sqrt{ALT\;\left(U/L\right)}} \end{array}$$

$$\begin{array}{l} APRI=\frac{AST\;\left(U/L\right)/ULN\;\left(U/L\right)}{PLT\;\left(1000\;cells/\;\mu \;L\right)}\times 100 \end{array}$$

$$\begin{array}{l} \begin{array}{l} \begin{array}{lll} & Frons\;\;\;index=7.811-3.131\times ln\left[PLT\;\left(1000\;cells/\;\mu \;L\right)\right]\; & +0.781\times ln\left[GGT\;\left(U/L\right)\right]\; \end{array}\;+3.467\times ln\left[age\;\left(years\right)\right]-0.014\times TC\;\left(mg/dL\right)\; \end{array} \end{array}$$

where AST = 37 U/L (male), AST = 31 U/L (female) used as the upper limit of normal for the 1999 to 2000 cycle, and AST = 33 U/L used as the upper limit of normal for 2001 to 2006 cycle.

#### 2.2.3. Covariates

Demographic variables include age, gender, race/ethnicity, education level, and family income level. Age was categorized as 20 to 39, 40 to 59, and 60+. The race categories were Hispanic, non-Hispanic White, and other races (including other non-Hispanic and multiracial). Education level was classified as less than high school, high school graduate or GED, some college or above. Family income levels were categorized as low (poverty income ratio [PIR] < 1.3), middle (1.3 ≤ PIR < 3.5), and high (PIR ≥ 3.5) based on the PIR. Physical activity was classified as inactivate, moderate, and vigorous based on whether the participants had been strenuously or moderately active in the past 30 days. Participants were categorized as under/normal weight (BMI < 25), overweight (25 ≤ BMI < 30), and obese (BMI ≥ 30). All participants were asked if smoked at least 100 cigarettes in their lifetime and now smoke cigarettes. Those who self-reported not having smoked 100 cigarettes in life were never smokers, those who had smoked more than 100 cigarettes and had quit were defined as former smokers, and current smokers were defined as having smoked more than 100 cigarettes and were still smoking at the time of the survey. According to the converted daily alcohol consumption, alcohol intake was classified into 1 to 3 drinks/d, 3 to 5 drinks/d, and more than 5 drinks. Participants with glycohemoglobin concentration ≥ 6.5% or fasting plasma glucose ≥ 126 mg/dL or who were informed by their physician that had diabetes or were using insulin or diabetic pills were diagnosed with diabetes. Continuous variables include serum folate, vitamin B_12_, total cholesterol, and high-density lipoprotein, values of which were obtained by blood sample testing.

### 2.3. Statistical analysis

We used weighted linear regression (continuous variables) and weighted chi-square tests (categorical variables) to compare baseline characteristics of participants between ALD and non-ALD or low fibrosis risk and intermediate-high groups fibrosis risk. Continuous variables were represented as means ± SD, and categorical variables were represented as frequencies (%). We used the weighted multivariate logistic regression models to estimate the relationship between serum Hcy and advanced hepatic fibrosis in ALD participants and expressed them as odds ratios (ORs) and 95% confidence intervals. Multiple models were adjusted for the main confounding factors, and serum Hcy was also categorized into tertiles. We fitted the restricted cubic spline (RCS) to further elucidate this association. In addition, we performed subgroup analyses stratified by gender, physical activity, and BMI. The area under the receiver operating characteristic curve was used to reveal the diagnostic performance of Hcy in assessing advanced hepatic fibrosis. All statistical analyses were performed using R (version 4.3.1) and *P* < .05 indicated statistical significance.

## 3. Results

### 3.1. Baseline characteristics of participants

The study included a total of 10,033 participants from NHANES 1999 to 2006. According to the criteria used to define ALD in this study, 496 participants were identified as ALD (Fig. 1). The demographics and baseline characteristics are presented in Table 1. The weighted prevalence of ALD was 5.31%. Compared to non-ALD participants, participants with ALD were more likely to be middle aged men, less educated, inactive physical activity, higher BMI levels and current smoking rates. ALD participants had higher serum Hcy levels, lower serum folate levels, and no significant difference in vitamin B_12_ levels compared with non-ALD participants.

**Table 1:** Baseline characteristics of participants in NHANES 1999 to 2006.

Characteristics	Overall	Non-ALD	ALD	*P*-value
n (%)	10,033 (100%)	9537 (94.69%)	496 (5.31%)	
Age (yr), n (%)				.038
20–39	4144 (43.07%)	3967 (43.23%)	177 (40.22%)	
40–59	3196 (39.63%)	2991 (39.29%)	205 (45.72%)	
60+	2693 (17.29%)	2579 (17.47%)	114 (14.06%)	
Gender, n (%)				<.001
Male	5333 (52.29%)	4941 (50.86%)	392 (77.73%)	
Female	4700 (47.71%)	4596 (49.14%)	104 (22.27%)	
Race/ethnicity, n (%)				.190
Hispanic	2571 (12.33%)	2446 (12.46%)	125 (10.10%)	
Non-Hispanic White	5496 (75.48%)	5225 (75.31%)	271 (78.44%)	
Other race	1966 (12.19%)	1866 (12.23%)	100 (11.47%)	
Education level, n (%)				<.001
Less than high school	2474 (15.18%)	2318 (14.89%)	156 (20.33%)	
High school graduate or GED	2351 (24.59%)	2214 (24.31%)	137 (29.53%)	
Some college or above	5208 (60.24%)	5005 (60.80%)	203 (50.14%)	
Income level, n (%)				.325
Low	2066 (14.66%)	1949 (14.60%)	117 (15.82%)	
Middle	3507 (32.60%)	3338 (32.55%)	169 (33.55%)	
High	3794 (47.05%)	3623 (47.26%)	171 (43.34%)	
Missing	666 (5.68%)	627 (5.59%)	39 (7.29%)	
Physical activity, n (%)				.003
Inactivate	3663 (29.98%)	3455 (29.73%)	208 (34.38%)	
Moderate	2943 (30.54%)	2792 (30.32%)	151 (34.30%)	
Vigorous	3427 (39.49%)	3290 (39.95%)	137 (31.32%)	
BMI group, n (%)				<.001
Under/normal weight	3302 (36.05%)	3164 (36.49%)	138 (28.18%)	
Overweight	3650 (34.77%)	3443 (34.18%)	207 (45.15%)	
Obese	3081 (29.18%)	2930 (29.32%)	151 (26.66%)	
Smoking status, n (%)				<.001
Never	4662 (46.42%)	4554 (47.78%)	108 (22.20%)	
Former	2763 (26.09%)	2628 (26.07%)	135 (26.46%)	
Current	2608 (27.49%)	2355 (26.15%)	253 (51.33%)	
Alcohol intake, n (%)				<.001
0–1 drinks/d	8250 (80.48%)	8250 (85.00%)	0 (0.00%)	
1–3 drinks/d	1421 (15.72%)	1158 (13.52%)	263 (54.96%)	
3–5 drinks/d	219 (2.34%)	83 (1.01%)	136 (26.09%)	
5 + drinks/d	143 (1.45%)	46 (0.47%)	97 (18.95%)	
Diabetes, n (%)				.932
No	9103 (93.21%)	8655 (93.20%)	448 (93.33%)	
Yes	930 (6.79%)	882 (6.80%)	48 (6.67%)	
Hcy (µmol/L)	8.49 ± 3.78	8.39 ± 3.59	10.42 ± 5.94	<.001
Folate, serum (nmol/L)	31.99 ± 22.50	32.17 ± 22.77	28.91 ± 16.79	<.001
Vitamin B_12_, serum (pmol/L)	389.24 ± 1150.85	390.06 ± 1176.39	374.60 ± 514.69	.230
ALT (U/L)	26.07 ± 19.31	25.26 ± 18.13	40.57 ± 30.58	<.001
AST (U/L)	24.92 ± 15.63	24.26 ± 13.50	36.68 ± 34.72	<.001
ALP (U/L)	69.38 ± 22.76	68.92 ± 22.29	77.49 ± 28.75	<.001
GGT (U/L)	29.70 ± 48.34	27.02 ± 33.29	77.48 ± 147.88	<.001
TBIL (mg/dL)	0.72 ± 0.32	0.72 ± 0.31	0.80 ± 0.34	<.001
TC (mmol/L)	5.22 ± 1.07	5.20 ± 1.06	5.61 ± 1.13	<.001
HDL (mmol/L)	1.39 ± 0.42	1.38 ± 0.41	1.48 ± 0.51	.451
FIB-4	0.89 ± 0.66	0.88 ± 0.63	1.07 ± 1.03	<.001
APRI	0.30 ± 0.27	0.29 ± 0.24	0.46 ± 0.58	<.001
Frons index	2.86 ± 1.66	2.83 ± 1.65	3.42 ± 1.78	<.001

For continuous variables, values were presented as means ± SD, and *P*-value was calculated by the weighted linear regression model. For categorical variables, data were shown as unweighted frequency counts and weighted percentages and weighted chi-square test was performed.

ALD = alcohol-related liver disease, ALP = alkaline phosphatase, ALT = alanine aminotransferase, APRI = AST-to-platelet ratio index, AST = aspartate aminotransferase, BMI = body mass index, FIB-4 = fibrosis-4 index, GED = general educational development, GGT = gamma glutamyl transaminase, Hcy = homocysteine, HDL = high-density lipoprotein cholesterol, NHANES = National Health and Nutrition Examination Survey, Non-ALD = non-alcoholic liver disease, TBIL = total bilirubin, TC = total cholesterol.

The weighted prevalence of those with intermediate-high risk of advanced hepatic fibrosis in ALD participants was 35.96%. Compared with ALD with low fibrosis risk, the 2 groups had statistically significant differences in age, physical activity, smoking status, alcohol consumption, and diabetes history. Serum Hcy and vitamin B_12_ were significantly increased, and serum folate levels were elevated in participants with advanced hepatic fibrosis intermediate-high risk (Table 2).

**Table 2:** Baseline characteristics of participants with ALD by advanced hepatic fibrosis status in NHANES 1999 to 2006.

Characteristics	Total	Advanced hepatic fibrosis		*P*-value
		Low fibrosis risk	Intermediate-high fibrosis risk	
n (%)	496 (100%)	291 (64.04%)	205 (35.96%)	
Age (yr), n (%)				<.001
20–39	177 (40.22%)	163 (57.44%)	14 (9.56%)	
40–59	205 (45.72%)	114 (38.38%)	91 (58.78%)	
60+	114 (14.06%)	14 (4.18%)	100 (31.67%)	
Gender, n (%)				.236
Male	392 (77.73%)	220 (75.76%)	172 (81.25%)	
Female	104 (22.27%)	71 (24.24%)	33 (18.75%)	
Race/ethnicity, n (%)				.541
Hispanic	125 (10.10%)	78 (10.94%)	47 (8.59%)	
Non-Hispanic White	271 (78.44%)	154 (76.98%)	117 (81.04%)	
Other race	100 (11.47%)	59 (12.08%)	41 (10.37%)	
Education level, n (%)				.504
Less than high school	156 (20.33%)	90 (21.62%)	66 (18.03%)	
High school graduate or GED	137 (29.53%)	88 (30.13%)	49 (28.46%)	
Some college or above	203 (50.14%)	113 (48.25%)	90 (53.51%)	
Income level, n (%)				.648
Low	117 (15.82%)	66 (16.76%)	51 (14.14%)	
Middle	169 (33.55%)	106 (34.66%)	63 (31.58%)	
High	171 (43.34%)	98 (42.14%)	73 (45.47%)	
Missing	39 (7.29%)	21 (6.44%)	18 (8.81%)	
Physical activity, n (%)				.021
Inactivate	208 (34.38%)	108 (29.58%)	100 (42.93%)	
Moderate	151 (34.30%)	86 (34.76%)	65 (33.49%)	
Vigorous	137 (31.32%)	97 (35.66%)	40 (23.58%)	
BMI group, n (%)				.849
Under/normal weight	138 (28.18%)	77 (27.84%)	61 (28.80%)	
Overweight	207 (45.15%)	124 (46.20%)	83 (43.28%)	
Obese	151 (26.66%)	90 (25.96%)	61 (27.92%)	
Smoking status, n (%)				.005
Never	108 (22.20%)	69 (25.74%)	39 (15.90%)	
Former	135 (26.46%)	63 (20.77%)	72 (36.59%)	
Current	253 (51.33%)	159 (53.48%)	94 (47.51%)	
Alcohol intake, n (%)				.009
1–3 drinks/d	263 (54.96%)	169 (60.01%)	94 (45.96%)	
3–5 drinks/d	136 (26.09%)	63 (21.42%)	73 (34.41%)	
5 + drinks/d	97 (18.95%)	59 (18.57%)	38 (19.63%)	
Diabetes, n (%)				.002
No	448 (93.33%)	271 (96.18%)	177 (88.24%)	
Yes	48 (6.67%)	20 (3.82%)	28 (11.76%)	
Hcy (µmol/L)	10.42 ± 5.94	9.46 ± 4.77	12.13 ± 7.30	<.001
Folate, serum (nmol/L)	28.91 ± 16.79	26.79 ± 14.52	32.69 ± 19.69	.002
Vitamin B_12_, serum (pmol/L)	374.60 ± 514.69	347.11 ± 146.88	423.56 ± 834.60	<.001
ALT (U/L)	40.57 ± 30.58	38.50 ± 19.92	44.27 ± 43.35	.168
AST (U/L)	36.68 ± 34.72	31.19 ± 11.83	46.45 ± 54.43	.002
ALP (U/L)	77.49 ± 28.75	75.89 ± 23.46	80.34 ± 36.20	.194
GGT (U/L)	77.48 ± 147.88	52.72 ± 40.17	121.57 ± 234.65	<.001
TBIL (mg/dL)	0.80 ± 0.34	0.77 ± 0.35	0.84 ± 0.33	.075
TC (mmol/L)	5.61 ± 1.13	5.75 ± 1.03	5.36 ± 1.25	.011
HDL (mmol/L)	1.48 ± 0.51	1.46 ± 0.48	1.50 ± 0.57	.545
FIB-4	1.07 ± 1.03	0.70 ± 0.26	1.73 ± 1.48	<.001
APRI	0.46 ± 0.58	0.34 ± 0.14	0.68 ± 0.91	<.001
Frons index	3.42 ± 1.78	2.46 ± 1.16	5.14 ± 1.35	<.001

For continuous variables, values were presented as means ± SD, and *P*-value was calculated by the weighted linear regression model. For categorical variables, data were shown as unweighted frequency counts and weighted percentages and weighted chi-square test was performed.

ALD = alcohol-related liver disease, ALP = alkaline phosphatase, ALT = alanine aminotransferase, APRI = AST-to-platelet ratio index, AST = aspartate aminotransferase, BMI = body mass index, FIB-4 = fibrosis-4 index, GED = general educational development, GGT = gamma glutamyl transaminase, Hcy = homocysteine, HDL = high-density lipoprotein cholesterol, NHANES = National Health and Nutrition Examination Survey, TBIL = total bilirubin, TC = total cholesterol.

### 3.2. Association between Hcy and advanced hepatic fibrosis in ALD

**Figure 2.**
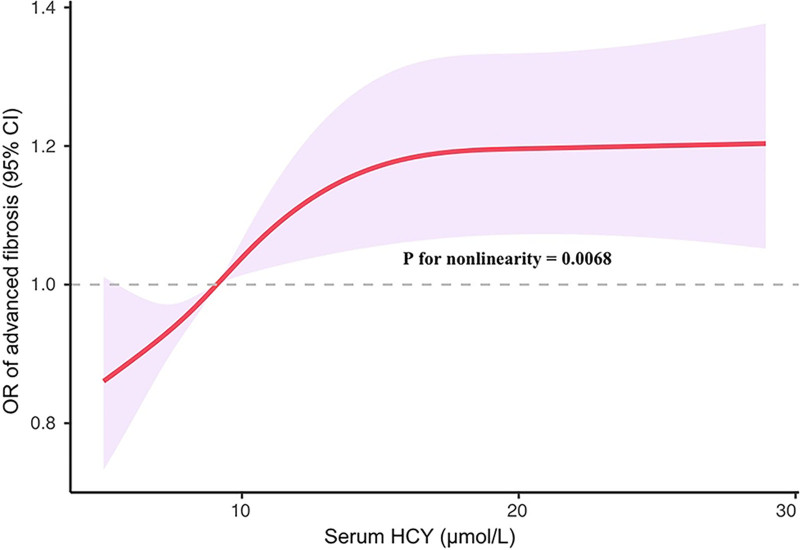
Restricted cubic spline (RCS) plot of the association between Hcy and advanced hepatic fibrosis. RCS regression was adjusted for age, gender, race/ethnicity, education level, physical activity, BMI, smoking status, alcohol intake, diabetes, serum folate, serum vitamin B_12_, TC, and HDL (model 3). The red solid line represents the odds ratios and the purple shadow area is expressed as the 95% confidence interval. BMI = body mass index, CI = confidence interval, Hcy = homocysteine, HDL = high-density lipoprotein, OR = odds ratio, RCS = restricted cubic spline, TC = total cholesterol.

Used multivariate logistic regression models to assess the association between Hcy and advanced hepatic fibrosis (Table 3). After adjusting for all confounding factors, we observed an association between serum Hcy and the risk of advanced hepatic fibrosis for ALD (OR = 1.07, 95% CI, 1.01–1.12, *P* = .018). ALD participants with the highest tertile of serum Hcy had significantly higher odds of developing advanced hepatic fibrosis than the lowest 1 (OR = 3.36, 95% CI, 1.34–8.43, *P* for trend = .011), indicating that higher levels of serum Hcy were positively correlated with advanced hepatic fibrosis risk. To further reveal the correlation between Hcy and the risk of advanced hepatic fibrosis, the RCS curve fitting was performed (Fig. 2). The multivariate-adjusted RCS curve showed that there was a nonlinear association between serum Hcy levels and the risk of liver fibrosis (*P* for nonlinearity = .0068), the risk of liver fibrosis increased progressively with increasing serum Hcy levels when Hcy was at <16.96 µmol/L.

**Table 3:** Association between Hcy and risk of advanced hepatic fibrosis in ALD, NHANES 1999 to 2006.

	Model 1 OR (95% CI)	Model 2 OR (95% CI)	Model 3 OR (95% CI)
Hcy (µmol/L)	1.09 (1.02, 1.17)*	1.06 (1.01, 1.10)*	1.07 (1.01, 1.12)*
Hcy (µmol/L)			
Tertile 1 (6.88 ± 0.92)	Reference	Reference	Reference
Tertile 2 (9.09 ± 0.63)	1.98 (1.21, 3.24)*	1.36 (0.66, 2.81)	1.35 (0.62, 2.93)
Tertile 3 (15.33 ± 8.16)	4.17 (2.34, 7.43)†	2.17 (1.03, 4.57)*	3.36 (1.34, 8.43)*
*P* for trend	<.001	.041	.011

Model 1: no covariates were adjusted. Model 2: age, gender, and race/ethnicity were adjusted. Model 3: age, gender, race/ethnicity, education level, physical activity, BMI, smoking status, alcohol intake, diabetes, serum folate, serum vitamin B_12_, TC, and HDL were adjusted.

ALD = alcohol-related liver disease, BMI = body mass index, CI = confidence interval, Hcy = homocysteine, HDL = high-density lipoprotein cholesterol, NHANES = National Health and Nutrition Examination Survey, OR = odd ratio, TC = total cholesterol.

* *P* < .05.

† *P* ≤ .001.

### 3.3. Subgroup analyses

**Figure 3.**
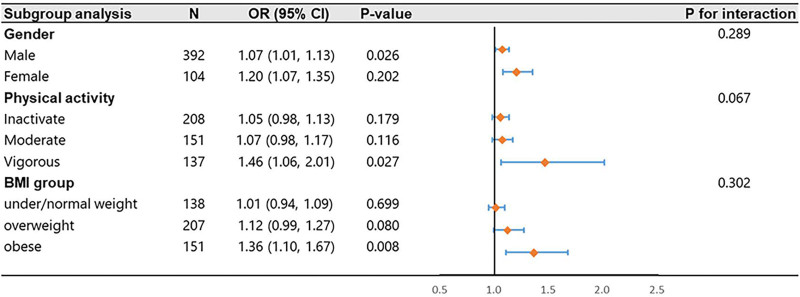
The subgroup analysis between Hcy and advanced hepatic fibrosis. Age, gender, race/ethnicity, education level, physical activity, BMI, smoking status, alcohol intake, diabetes, serum folate, serum vitamin B_12_, TC, and HDL were adjusted (model 3). In each subgroup analysis, the model was not adjusted for the stratification variable itself. BMI = body mass index, CI = confidence interval, Hcy = homocysteine, HDL = high-density lipoprotein, OR = odds ratio, TC = total cholesterol.

Subgroup analysis was conducted based on stratified factors such as gender (male, female), physical activity (inactivate, moderate, vigorous), and BMI (under/normal weight, overweight, obese; Fig. 3). The results showed that the associations between serum Hcy and advanced hepatic fibrosis were more significant in men (OR = 1.07, 95% CI, 1.01–1.13, *P* = .026), vigorously physically active (OR = 1.46, 95% CI, 1.06–2.01, *P* = .027), and obese participants (OR = 1.36, 95% CI, 1.10–1.67, *P* = .008). However, the subgroups’ interaction effect was insignificant (*P* for interaction >.05).

### 3.4. Diagnostic performance of Hcy for advanced hepatic fibrosis in ALD

In all ALD participants, the area under the working characteristic curve of serum Hcy for estimating the risk of advanced hepatic fibrosis was 0.686 (95% CI, 0.639–0.733), and the optimal cutoff value of Hcy to detect advanced hepatic fibrosis was 9.27 µmol/L, with a sensitivity of 66.3% and a specificity of 65.6%.

## 4. Discussion

This study aimed to investigate the association between serum Hcy levels and advanced hepatic fibrosis in ALD patients. In this cross-sectional study based on the population from the NHANES database, we observed significantly elevated serum Hcy levels in participants with ALD. Serum Hcy levels were significantly and positively associated with the risk of advanced hepatic fibrosis, and the association was nonlinear. Hcy was independently associated with advanced hepatic fibrosis risk in ALD participants.

Oxidative stress and inflammatory responses are widely involved in the formation and development of ethanol-induced liver fibrosis. Under the influence of reactive oxygen species and persistent inflammation repeatedly activate Kupffer cells to secrete various proinflammatory cytokines and chemokines, HSCs are activated and produce a large amount of collagen and extracellular matrix components, which is a key link in the progression of early alcoholic liver disease to advanced hepatic fibrosis and cirrhosis.^[16]^ Hcy is an important intermediate product of methionine methylation, and 85% of Hcy transsulfuration occurs in the liver, making the liver an important metabolizing organ for Hcy. Dietary folate is successively converted to dihydrofolate and tetrahydrofolate by dihydrofolate reductase, and then converted by a series of enzymatic reactions involving vitamin B_6_, vitamin B_12_, and betaine to 5-methyltetrahydrofolate, which participates in the methionine cycle as a methyl donor. Among them, folate and vitamin B_12_, as cofactors necessary for the Hcy metabolic pathway, are the main factors affecting serum Hcy levels.^[10]^ Chronic heavy drinking can lead to folate and vitamin B_12_ deficiencies in hepatocytes, ethanol directly inhibits the activity of methionine synthase which is a key enzyme in the methionine cycle, and the alternative pathway of hepatic betaine is not sufficiently compensated for, the above synergistic effects result in markedly impaired Hcy remethylation, contributes to Hcy accumulation and elevated serum Hcy levels.^[17]^ Hyperhomocysteinemia can promote an inflammatory response, enhance oxidative stress, and activate endoplasmic reticulum stress to induce lipid synthesis, apoptosis, and inflammation in liver cells.^[18]^ Zou et al found that Hcy promotes HSC proliferation by eliciting a transient reactive oxidative species formation and activation of nicotinamide adenine dinucleotide phosphate (NADPH) enzymes, a process that involves the phosphorylation of various protein kinases and the induction of multiple oxidative stress pathways, which explains the mechanism of hyperhomocysteinemia mediated hepatic fibrosis.^[19]^ García-Tevijano et al observed a marked reduction in the expression of main genes related to Hcy metabolism in patients with alcoholic cirrhosis, which correlated with the severity of liver fibrosis.^[20]^ Hcy directly induces the expression of the tissue inhibitor of metallo-proteinases-1 and α1(I) procollagen expression in liver cells and HSCs, leading to the accumulation of extracellular matrix and induces or exacerbates liver fibrosis.^[21]^

Our study indicated that serum Hcy levels were elevated, and serum folate levels were lower in ALD participants compared to non-ALD controls, indicating ALD patients have folate deficiency and high Hcy levels, similar to previous research findings.^[22,23]^ Studies have shown a significant negative correlation between serum Hcy and serum folate. Chronic alcoholics with liver injury have reduced serum folate values, and the incidence of hyperhomocysteinemia is significantly increased.^[24]^ Our results also showed that ALD participants with intermediate-high risk of advanced hepatic fibrosis exhibited higher Hcy levels compared to low fibrosis risk, while their serum folate and vitamin B_12_ levels were also higher. In addition, Llibre-Nieto et al classified alcoholic cirrhosis patients according to the Child-Pugh score and found that vitamin B_12_ levels were significantly higher in Child-Pugh class C patients than in Child-Pugh class A and B patients.^[25]^ This may be related to the excessive release of vitamin B_12_ from damaged hepatocytes and reduced uptake from serum.^[26]^ Bosy-Westphal et al measured plasma Hcy and its metabolism-dependent cofactors folate and vitamin levels in 43 patients with biopsy-proven cirrhosis, they found that plasma Hcy concentrations were elevated in patients with alcoholic cirrhosis, plasma vitamin B_12_ concentrations increased as liver function deteriorated, and folic acid remained unchanged, Hcy concentrations did not correlate with plasma folate and vitamin B_12_ levels.^[27]^ Although slightly different from our results, both suggest that changes in Hcy levels in advanced liver disease cannot be explained by plasma levels of cofactors such as vitamin B_12_ and folate.

Our results supported an independent association between Hcy and advanced hepatic fibrosis in ALD, with a significant positive correlation between the 2, especially for men, vigorously physically active, and obese populations. Estradiol is the main factor contributing to gender differences in the progression of liver fibrosis.^[28]^ Experimental studies have revealed that estrogen can inhibit NF-kappaB-mediated inflammation through estrogen/ER signaling and may also exert antifibrotic effects by inhibiting HSC activation and proliferation.^[29,30]^ Estrogen, as a protective factor, may be associated with weakening the correlation between Hcy and advanced hepatic fibrosis in female ALD patients. Fatigue is the most common symptom in patients with chronic liver disease, and recent studies have shown a strong association between fatigue and the severity of liver inflammation, in patients with chronic liver disease with fatigue symptoms, the inflammation grades and liver fibrosis stages are significantly increased.^[31]^ Therefore, we think that ALD patients who strive to maintain high levels of physical activity have an increased burden of fatigue and may be at increased risk of worsening liver disease.^[32]^ Recent evidence suggests that obesity and alcohol exhibit synergistic effects on the onset and progression of liver injury, with important effects for hepatocellular lipid accumulation and damage, liver inflammation and fibrosis,^[33,34]^ and obesity may be an independent predictor of liver-related mortality in patients with ALD.^[35]^ We have reason to believe that the association between Hcy and advanced hepatic fibrosis is more significant in obese ALD patients.

Our study suggested that Hcy has certain diagnostic value in assessing the risk of advanced hepatic fibrosis in ALD, and could serve as a predictive indicator and therapeutic entry point for early ALD patients. Chen et al observed high levels of hepatic matrix metalloproteinase-2, hepatic cytochrome 2E1, and significant liver fibrosis in long-term ethanol-feeding rats, which may be related to Hcy mediation, while combined treatment of folate and vitamin B_12_ can delay the progression of liver fibrosis by lowering serum Hcy levels.^[36]^ As an important human nutrient and dietary supplement, Betaine plays an important role in maintaining adequate liver methionine supply and regulating Hcy concentration. Several studies have shown that betaine can alleviate liver steatosis, inflammation, and fibrosis to improve ALD, the process involves reducing Hcy concentration by restoring impaired methionine metabolism.^[37,38]^ Vatsalya et al showed that hyperhomocysteinemia was strongly related to elevated alcohol withdrawal rates and liver injury, and hyperhomocysteinemia may be an indicative marker for early ALD patients accompanied by alcohol use disorders.^[39]^ However, there is a lack of clinical trials to demonstrate the benefits of folate, vitamin B_12_, and betaine in the treatment of ALD, prospective studies are still needed to evaluate the safety and effectiveness of these therapies, and to provide more clinical rationales for Hcy to become an important predictive marker for assessing the risk of liver fibrosis.

The strengths of this study were to comprehensively assess the relationship between serum Hcy and liver fibrosis in ALD for the first time in a large sample of data and to adjusted for confounding factors as much as possible to make our research results more convincing. However, our study also has limitations. Firstly, although the NHANES mobile examination centers interviewer staff administered a detailed alcohol use questionnaire to all participants, their responses may be biased by recall error. We used FIB-4, APRI, and Frons indices to evaluate advanced hepatic fibrosis in ALD subjects and classify its severity, despite these noninvasive indicators have been widely used for the assessment and diagnosis of liver fibrosis caused for various reasons, they have lower sensitivity and validity than the Fibroscan examination and liver biopsy.^[40,41]^ Due to limited Hcy detection, this study only collected data from NHANES 1999 to 2006. Furthermore, due to the cross-sectional study design, we are unable to determine the causal relationship between serum Hcy and advanced hepatic fibrosis and therefore require recent prospective cohort studies to elucidate this. In recent years, with the changes in people’s alcohol consumption, behavioral habits, and detection techniques, we speculate that the study results may differ slightly.

## 5. Conclusion

The results of our study suggested that serum Hcy levels were significantly and positively associated with an increased risk of advanced hepatic fibrosis in patients with ALD, especially in men, vigorously physically active, and obese populations. This association explains that Hcy is involved in the pathogenesis of ALD and provides a theoretical basis for Hcy to become a sensitive predictive marker for advanced hepatic fibrosis. Correcting Hcy metabolic disorders may become a new clinical therapeutic strategy for preventing and delaying the progression of liver fibrosis in ALD.

## Acknowledgments

The authors thank the NHANES teams for collecting and sharing datasets.

## Author contributions

**Conceptualization:** Cheng Ma, Xiaoqian Zhang.

**Data curation:** Cheng Ma, Wenxin Zhang.

**Formal analysis:** Cheng Ma, Jianzhou Duan.

**Investigation:** Cheng Ma, Hui Yang.

**Methodology:** Cheng Ma, Xiaoqian Zhang, Wenxin Zhang.

**Software:** Cheng Ma, Jianzhou Duan.

**Validation:** Cheng Ma, Hui Yang.

**Visualization:** Cheng Ma, Xiaoqian Zhang.

**Writing – original draft:** Cheng Ma.

**Writing – review & editing:** Cheng Ma, Hui Yang.
